# Soft Robotic Textiles for Adaptive Personal Thermal Management

**DOI:** 10.1002/advs.202309605

**Published:** 2024-03-26

**Authors:** Xiaohui Zhang, Zhaokun Wang, Guanghan Huang, Xujiang Chao, Lin Ye, Jintu Fan, Dahua Shou

**Affiliations:** ^1^ Future Intelligent Wear Centre School of Fashion and Textiles The Hong Kong Polytechnic University Kowloon 999077 China; ^2^ Research Centre of Textiles for Future Fashion The Hong Kong Polytechnic University Kowloon 999077 China; ^3^ Research Institute for Intelligent Wearable Systems The Hong Kong Polytechnic University Kowloon 999077 China; ^4^ State Key Laboratory of Precision Electronic Manufacturing Technology and Equipment Guangdong University of Technology Guangzhou 510006 China; ^5^ School of Mechanical Engineering Northwestern Polytechnical University Xi'an 710072 China; ^6^ School of System Design and Intelligent Manufacturing (SDIM) Southern University of Science and Technology Shenzhen 518055 China

**Keywords:** adaptive thermal Insulation, low boiling point fluid, personal thermal management, phase transition, soft robotic textile

## Abstract

Thermal protective textiles are crucial for safeguarding individuals, particularly firefighters and steelworkers, against extreme heat, and for preventing burn injuries. However, traditional firefighting gear suffers from statically fixed thermal insulation properties, potentially resulting in overheating and discomfort in moderate conditions, and insufficient protection in extreme fire events. Herein, an innovative soft robotic textile is developed for dynamically adaptive thermal management, providing superior personal protection and thermal comfort across a spectrum of environmental temperatures. This unique textile features a thermoplastic polyurethane (TPU)‐sealed actuation system, embedded with a low boiling point fluid for reversible phase transition, resembling an endoskeleton that triggers an expansion within the textile matrix for enhanced air gap and thermal insulation. The thermal resistance improves automatically from 0.23 to 0.48 Km^2^ W^−1^ by self‐actuating under intense heat, exceeding conventional textiles by maintaining over 10 °C cooler temperatures. Additionally, the knitted substrate incorporated into the soft actuators can substantially mitigate convective heat transfer, as evidenced by the thermal resistance tests and the temperature mapping derived from numerical simulations. Moreover, it boasts significantly increased moisture permeability. The thermoadaptation and breathability of this durable all‐fabric system signify considerable progress in the development of protective clothing with high comfort for dynamic and extreme temperature conditions.

## Graphical abstract

An innovative soft robotic textile is developed for passive, adaptive thermal management, making it ideal for a wide range of applications, including firefighters' gear and protective clothing. This intelligent textile autonomously adjusts thermal insulation using a flexible and robust actuation system, optimizing both protection and comfort across dynamically changing thermal conditions. This groundbreaking clothing technology also offers superior breathability.

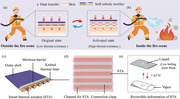

## 1 Introduction

Heat‐related risks are increasingly prevalent for individuals in high‐temperature environments, notably firefighters who often confront unpredictable blazes. According to the U.S. Fire Administration, approximately 15200 injuries and 3500 fatalities occurred in 2020 as a result of firefighting efforts, which included the loss of 102 firefighters,^[^ ^1^ ^]^ underscoring the critical role of thermal protective clothing (TPC). TPC acts as a thermal barrier against heat, avoiding thermal injuries by reducing direct exposure to intense heat and flames, thus diminishing the risk of burns and death.^[^ ^2^ ^]^ Consequently, there is an imperative need to advance the development of high‐performance TPC that can effectively manage body temperature and shield wearers from extreme temperatures and the dangers of excessive heat stress and stroke.^[^ ^3^ ^]^

Traditional TPC is often constructed from multi‐layered fabrics with a thick, bulky thermal liner, wherein greater insulation is achieved by increasing the thickness of the liner.^[^ ^4^, ^5^ ^]^ However, this conventional approach results in heavier garments with reduced breathability, which consequently hampers the mobility and efficiency of the wearers.^[^ ^6^ ^]^ Recent research efforts have focused on incorporating aerogel into TPC to enhance thermal protection while maintaining a lighter weight.^[^ ^7^, ^8^, ^9^, ^10^ ^]^ The aerogel is renowned for its exceedingly low density, high porosity, and low thermal conductivity, becoming an excellent insulator.^[^ ^10^, ^11^, ^12^ ^]^ Despite these benefits, fabrics integrated with aerogel suffer from fixed thermal conductivity that struggles to respond quickly to changes in temperature. Moreover, the presence of aerogel can significantly impede moisture transfer, compromising the comfort of the garment. In pursuit of more dynamic solutions, there has been a growing interest in developing intelligent fabrics that adapt to environmental temperatures through the use of temperature‐sensitive materials, such as phase change materials (PCM). PCM can absorb and release a significant amount of latent heat during their phase transitions, thereby shielding the human body from external heat fluxes.^[^ ^13^, ^14^, ^15^ ^]^ Various types of PCM have been incorporated into TPC to enhance wearer comfort and safety.^[^ ^16^, ^17^, ^18^ ^]^ Furthermore, combining both aerogel and PCM with traditional fabrics has also improved thermal insulation properties.^[^ ^19^, ^20^, ^21^ ^]^ However, limitations persist, particularly concerning the short duration of thermal protection and high moisture resistance inherent in these designs.^[^ ^22^ ^]^ An alternative strategy is based on leveraging the lower thermal conductivity of still air.^[^ ^23^, ^24^ ^]^ Creating an air gap between fabric layers to create a buffer microclimate can significantly boost thermal insulation. Another innovative approach includes integrating shape memory alloys (SMAs) with TPC to regulate the air gap for enhanced thermal insulation.^[^ ^25^, ^26^ ^]^ Such garments provide adequate insulation under extreme heat, while also offering less thermal resistance to maintain comfort in normal conditions.^[^ ^25^, ^27^ ^]^ Spring‐like SMAs, which transform from flat to 3D states,^[^ ^28^ ^]^ act as actuators in response to certain temperatures, expanding the air gap between fabric layers to block heat transfer. Nevertheless, SMAs typically exhibit a one‐way shape memory effect and do not easily return to their original shape once the temperature normalizes.^[^ ^29^ ^]^ Additional challenges include controlling the position, size, and stability of the stiff SMA‐based actuators within the soft fabric system.

An ideal thermally adaptive textile should exhibit fast responsive thermal behavior, retaining distinct configurations across varying temperatures to maintain consistent thermal comfort. This must be achieved without compromising flexibility or moisture permeability for personal thermal management. In this paper, we present a new concept of a soft robotic textile (SRT), engineered to provide dynamic and adaptive thermal insulation. This unique idea has been achieved through the integration of smart thermal actuators (STAs) which incorporate low boiling point fluids sealed within the system. These actuators function by modulating fabric thickness and structure, precisely regulating the air space between textile layers to adjust the thermal insulation. Thermally activated actuators have recently gained increasing attention as the chosen low boiling point fluid undergoes a dramatic volumetric expansion upon phase change from liquid to vapor.^[^ ^30^, ^31^ ^]^ This effect can result in the inflation of the actuators, which, in turn, forces the textile layers apart, generating an insulating, structured air gap that impedes heat conduction. To further enhance performance, we have also employed a knitted fabric seamlessly replete with channels, serving as both the thermal liner and the encapsulation framework for the intelligent thermal actuators. This innovative all‐fabric design not only provides exceptional thermal insulation and structural stability but also exhibits a reversible response to temperature fluctuations.

## 2 Results and Discussion

**Figure 1:**
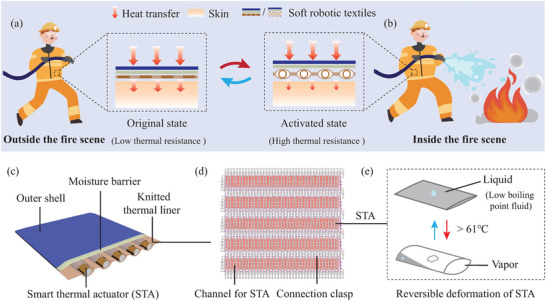
Design concept of thermally adaptive soft robotic textiles. a) Under normal conditions, the SRT's slim profile and reduced thermal resistance facilitate the transfer of heat and moisture away from the skin. b) Upon exposure to a fire environment, the SRT activates to provide enhanced thermal protection maintaining lightness and breathability. Specifically, outside fire events, SRT layers deflate for low thermal resistance; when activated in a fire scenario, the layers separate, creating an insulating air gap that impedes heat transfer. c) Illustration of the SRT featuring a flame‐resistant outer layer, a breathable moisture barrier, a knitted thermal liner, and an STA filled with a low boiling point fluid. d) Construction specifics of the knitted thermal liner with embedded STAs. e) Phase transition of a low boiling point fluid within the STA, characterized by significant volume change: in ambient temperatures, the fluid remains in a liquid state; as temperatures rise, the fluid vaporizes, and then reverts into a liquid upon cooling.

**Figure** **1** illustrates an SRT that can passively adjust its thickness for adaptive thermal insulation in different temperature environments, utilizing a smart thermal actuator (STA) based on a low boiling point fluid. This unique thermally protective textile is designed to maintain thermal comfort under temperate conditions by employing a thin fabric with low thermal resistance. In contrast, it can increase the air gap between fabric layers, which boosts thermal resistance and blocks heat during exposure to higher temperatures. The conventional thermal protective textiles possess a static structure with fixed thermal resistance. Consequently, firefighters must wear such garments before entering a fire scene, which can impede both heat and moisture transfer from the skin to the surrounding air. This can cause discomfort due to overheating, particularly in summer. Upon entering a high‐temperature environment like a fire scene, the bulk and weight of traditional protective clothing can restrict movement and potentially decrease work efficiency. The design of the SRT shown in Figure 1a,b addresses this issue. When outside the fire scene, the SRT maintains a lightweight and highly thermally conductive state due to its thin, compact fabric structure. However, when a firefighter enters the fire scene and the ambient temperature rises, the STA activates, causing an increase in fabric thickness and thermal resistance. Should the firefighter exit the hot conditions, the lower temperature triggers the STA to revert to its original state, resulting in a reversible deformation of the SRT. Therefore, the SRT can remain thin with low thermal resistance in regular temperatures to ensure comfort, yet automatically adapt to protect the wearer from heat by enlarging the air gap for improved thermal resistance. It then reverts to being thin as the temperature drops, allowing one piece of clothing to be suitable for a wide range of temperatures. Figure 1c presents the structure of the SRT, which includes a flame‐retardant outer shell, a breathable waterproof moisture barrier, and a porous knitted thermal liner showcased in Figure 1d. The system also features thickness‐adjustable STAs depicted in Figure 1e. The thermal liner has integrated channels that house and secure the STAs, along with clasps that connect these channels. The fluid within the STA has a low boiling point of 61 °C, enabling it to undergo reversible phase changes from liquid to vapor in response to temperature fluctuations.

**Figure 2:**
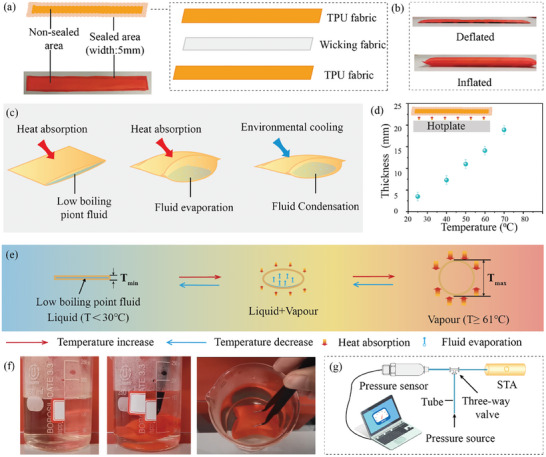
Configuration and working mechanism of the STA. a) Structure of the STA. A rectangular pouch (30 mm × 300 mm) is constructed from heat‐sealing TPU fabric with an inner layer of cotton fabric, serving as a wicking material to contain a low boiling point fluid. b) The STA depicted in both fully deflated and inflated states. c) Illustration of the reversible deformation of the STA during phase changes between liquid and vapor: At ambient temperature, the contained fluid remains in a liquid state; upon heating, the fluid absorbs heat and begins to evaporate, whereas cooling induces condensation of the vapor. d) Correlation between temperature and thickness when the STA is heated. e) Cross‐sectional view of the STA during the deformation process in different temperatures. With increasing temperature, the fluid transitions to a state of evaporation due to sustained heat absorption against a strong environmental cooling effect, maintaining a mixed liquid‐vapor phase. As the temperature surpasses the fluid's boiling point, the STA inflates completely, filled with vapor, resembling a circular cross‐section. f) Leakage assessment for the STA as submerged in hot water. g) Evaluation of the maximum pressure endurance of the STA.

The heat‐sealed nylon fabric, commercially sourced and featuring an impermeable thermoplastic polyurethane (TPU) coating on one side, was employed to construct the STA. The TPU‐coated side underwent a heat‐sealing process using thermal bonding technology, which promises scalability for industrial production. The details of the manufacturing process are depicted in Figure S1 (Supporting Information). The fabricated STA takes the form of a rectangular pouch (30 mm × 300 mm) with sealed edges that are 5 mm in width. The non‐sealed region is constructed as an air chamber with a sizable volume of vapor produced during fluid evaporation, embedded with a layer of wicking fabric for storage of the boiling fluid. As illustrated in **Figure** **2**a, the STA comprises three layers, with a cotton fabric interlayer serving as the wicking material, adhered between the layers of heat‐sealed nylon fabric. This wicking layer is capable of absorbing and sustaining liquid, thereby facilitating the uniform distribution of a low boiling point fluid. Figure 2b presents the STA in both deflated and inflated states. It appears flat when deflated but exhibits an elevated surface and increased height upon full inflation under the temperature stimuli. The deformation mechanism underlying the STA's operation is demonstrated in Figure 2c: the low boiling point fluid absorbs ambient heat, vaporizes, and consequently inflates the STA. Conversely, a reduction in ambient temperature causes the vapor to release heat and revert to a liquid state. Then the STAs were subjected to temperature‐responsive deformation on a hotplate, with the process captured in Movie S1 (Supporting Information). This cycle was repeated multiple times (over 20 times) to assess the STA's reversible functionality, indicating that within a limited range of cyclic usage, the decrease in performance could be negligible.

The correlation between the hotplate temperature and the STA's thickness is shown in Figure 2d, where the employed width is 30 mm, and the maximum observed thickness reached 19.1 mm. The correlation between the unsealed area's width and the STA's maximum thickness can be discerned from Equation S2 (Supporting Information). Our distinct design considerably mitigates the impact of the non‐stretchable nylon fabric STA on thickness enhancement. During the phase change, the STAs transition from a flat state to a cubic cylinder‐like state, thereby increasing the thickness of both the STA and garment. It is noted that we have incorporated the nylon fabric actuators into stretchable knitted fabric substrates. As a result, the non‐stretchability of the local actuators will not impact the overall stretchability of the soft robotic textile. Figure 2e and Movie S2 (Supporting Information) showcase the cross‐section of the STA during deformation, highlighting its transition from a flat, thin liquid state at room temperature to a mixed liquid‐vapor state and eventually to a full vapor state with maximal thickness upon heat application. Theoretical modeling of the STA's behavior is portrayed in Figure S2 (Supporting Information), suggesting that the maximum thickness achievable by the STA is dictated by the initial breadth of its air chamber.

To ascertain the sealing strength and ensure no leakage of vapor or liquid, a leakage test was conducted. The outcomes, visualized in Movie S3 (Supporting Information), involved submerging a small STA in hot water (exceeding 80 °C) to observe bubble formation—or lack thereof—as indicative of seal integrity. Figure 2f illustrates the STA submerged in hot water, whereby heat absorption leads to fluid vaporization and a subsequent rise in the beaker's liquid level. A top‐down view confirms the fully inflated condition of the STA without any bubble formation, signifying the absence of vapor leakage. In an additional experimental setup, presented in Figure 2g, the STA demonstrated a capability to withstand pressures up to 1.96 MPa, showcasing its robustness for practical wear applications. As outlined in Supplementary Material, the sealed area can endure a pressure of 1.96 MPa at an equivalent temperature of 185.5 °C. Working beyond this temperature could lead to structural failures in the STA, including potential liquid and air leakage. Hence, we consider 185.5 °C as the STA's maximum operating temperature for the current prototype. Notably, the amount of low boiling point fluid applied for each STA was also crucial, which has been analyzed in the fourth section of Supplementary Materials. Smaller amounts of fluid may not fully inflate STA, leading it to operate sub‐optimally. Conversely, using too much fluid might increase the weight and cost of the STA, and could also result in higher internal pressure. This might potentially exceed the maximum safe pressure for the STA, as determined from the test results shown in Figure 2g. Therefore, it is significant to maintain a moderate amount of low boiling point fluid to ensure the STA operates at its full capacity and without risk of failure in practical applications. A volume of 1 mL of liquid was injected into the STA as mentioned earlier. The dual‐phase heating‐cooling test, leakage test, peak pressure endurance test, and calculations were all conducted to verify the STA's reliability and functionality under real‐world conditions.

**Figure 3:**
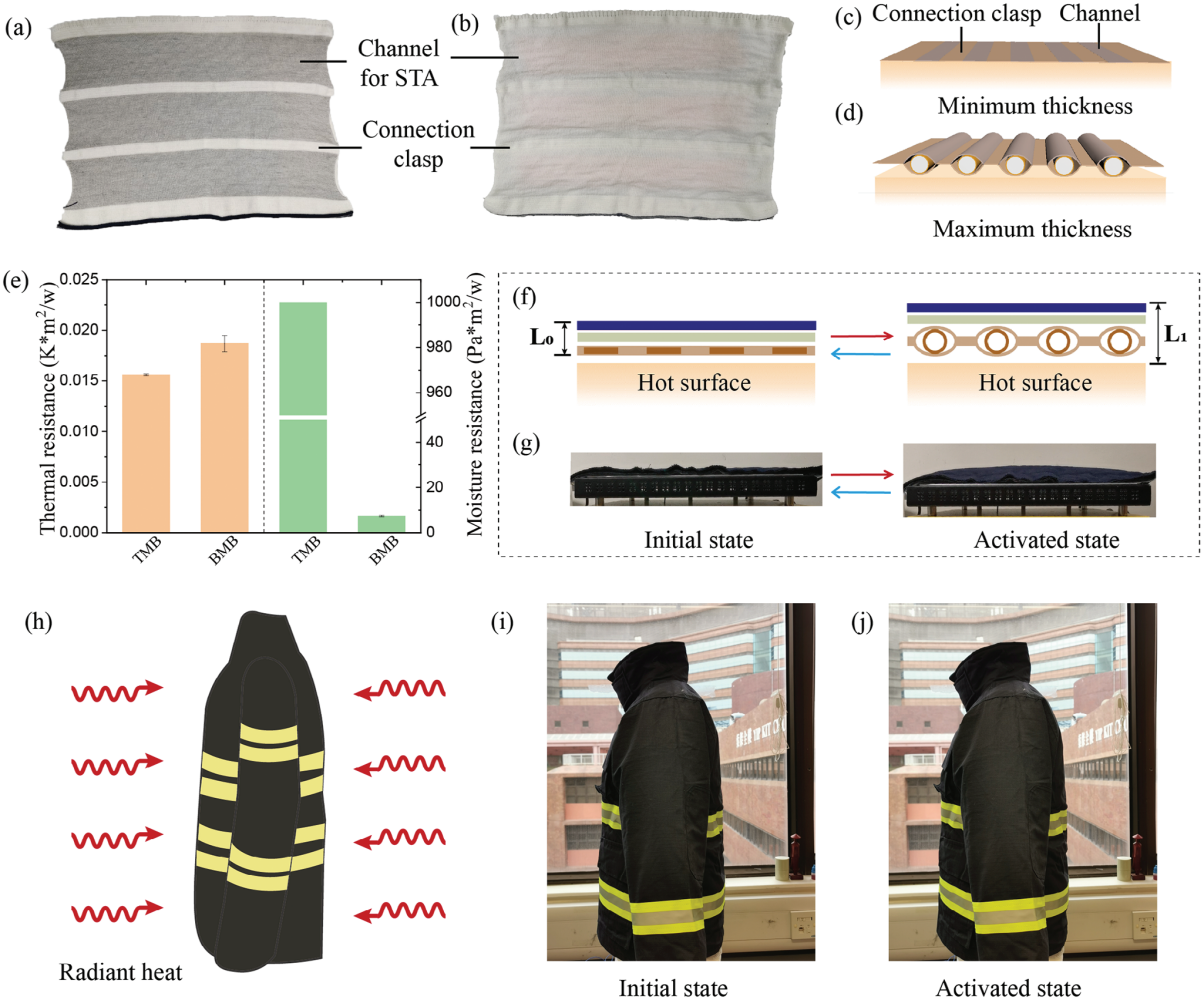
Thermoadaptation of STA embedded in a customized knitted fabric. a,b) Illustrations of the knitted thermal liner (240 mm × 340 mm) featuring a connecting clasp (width:15 mm) and a channel (width:60 mm) for STA. c) The STA within the knitted thermal liner depicted in a fully deflated state, exhibiting minimal thickness. d) The STA encapsulated in the knitted thermal liner shown in a fully inflated state, achieving maximal thickness. e) Comparison of thermal and moisture resistance between traditional liners and present SRTs. f) Cross‐sectional views of the SRT in the fully deflated and fully inflated states. g) Images of the SRT in its initial, inactive state progressing to the fully activated state. h) Schematic representation of an SRT‐based firefighter garment exposed to radiant heat. i) Side view of the garment in its initial state. j) Side view of the garment once thermally activated.

Subsequently, the STAs were integrated into textile systems in a seamless and robust pattern. **Figure** **3**a,b presents illustrations of the knitted thermal liner (240 mm × 340 mm) featuring channels designed for STAs and a fabricating connection clasp produced by a flat knitting machine. The channels designated for the STAs are depicted in grey, whereas the connection clasps are shown in white. The dimensions of both the channels and the connection clasps can be tailored by altering the number of knitting courses, facilitating compatibility with STAs of varying specifications. The width of the connection clasp mentioned herein is 15 mm and the channel is 60 mm, facilitating an effortless insertion process for the STAs. Each channel comprises a dual‐layer structure to encapsulate the STA, and the connection clasp serves to link these channels. Figure 3c,d illustrates diagrams of the thermal liner, knitted with integrated STAs, positioned on flat skin. When the STA is in a fully deflated state, the knitted thermal liner containing the STA remains flat with minimal thickness. Conversely, upon full inflation of the STA, the expanded structure forces the channels apart while elevating the connection clasp to the midpoint of the entire assembly, resulting in maximal thickness. Consequently, an air gap is formed and segmented by the connection clasp. Given that conventional thermal protective insulations are not or less permeable, a breathable moisture barrier was chosen to enhance thermal comfort performance without compromising waterproofness and breathability. Importantly, our design incorporates a connection clasp to prevent continuous STA arrangement, thus maintaining the textile's overall flexibility and stretchability. In essence, the strategically placed connection clasp not only preserves the textile's flexibility, but also facilitates moisture transfer between gauges, ensuring wearer comfort and fabric breathability. The thermal and moisture resistances of both the traditional and the newly proposed moisture barriers were evaluated, as depicted in Figure 3e. In comparison to the traditional moisture barrier, the proposed SRT offers similar thermal resistance yet significantly lower moisture resistance. The traditional barrier displayed a substantial moisture resistance (Ret) of over 350 Pa m^2^ W^−1^, ≈50 times greater than that of the breathable moisture barrier, which has a Ret value of 7.481 Pa m^2^ W^−1^ for supreme breathability. Figure 3f reveals the cross‐section of the SRT in both fully deflated and inflated states. The incorporation of the knitted thermal liner ensures that the STAs are held stably in place, preventing any displacement.

**Figure 4:**
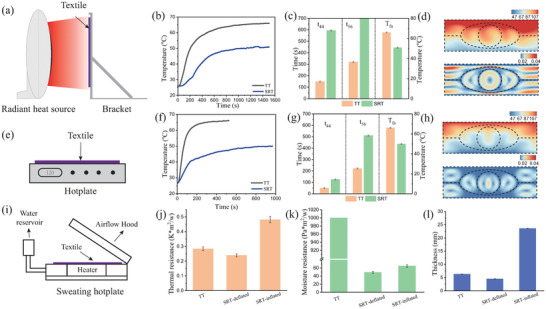
Experimental and Simulated Results of Thermal Analysis. a) Configuration of the radiant heat exposure test setup. b) Inner layer temperature profiles of traditional textiles (TT) and SRT under radiant heat. c) Duration required for reaching threshold temperatures of 44 and 56 °C, and the ultimate temperature attained during radiant heat exposure (Note: t_56_ = 700 s indicates the test duration to attain 56 °C). d) Simulated temperature and airflow velocity distribution in SRT subjected to radiant heat. e) Illustration of the hot surface contact test apparatus. f) Inner layer temperature profiles of TT and SRT during direct contact with a heated surface. g) Variations of time required to reach 44 and 56 °C upon contact with a hot surface. h) Simulation of temperature and airflow velocity distribution in SRT during hot surface contact. i) Set‐up for evaluating thermal and moisture resistance properties. j) Comparison of thermal resistance of TT, SRT in a deflated state, and SRT in an inflated state. k) Comparison of moisture resistance of TT, SRT in a deflated state, and SRT in an inflated state. l) Comparison of thickness for TT, SRT in a deflated state, and SRT in an inflated state.

The thermal mechanism of the proposed smart textile is intricately linked to the reversible phase transition of the low boiling point fluid contained within the actuators. When the ambient temperature is lower than the boiling point of the fluid, it remains in a thin layer state. At this stage, efficient thermal conduction both within the fluid and through the textile is undergone. Conversely, as the temperature rises above the boiling point of the fluid, the fluid transitions from the liquid state to vapor. This change in state results in a thickening of the fabric due to the expansion of the actuator, thereby creating more air gaps between fabric layers. As the still air has a particularly low thermal conductivity, this enhanced fabric thickness improves the thermal resistance of the textile. Besides, the creation of an air gap heightens the thermal convection process, particularly when there is a substantial temperature difference across the fabric. To mitigate this, we have incorporated insulating components—such as knitted fabric substrates with integrated connection clasps—to restrict it. Notably, when the temperature drops, the vapor returns to its liquid state. This dynamic phase transition process operates as a thermal switch, enabling the textile to adjust to varying temperature conditions and maintain an optimal level of thermal comfort for the wearers. The SRT thickness increases substantially upon full inflation from its initially reduced thickness in flat form, as demonstrated in Figure 3g. The SRT's transformation process is further demonstrated in Movie S4 (Supporting Information). We also subjected a manikin dressed in our SRT‐based garment to radiant heat on both sides, replicating an authentic fire scene (Figure 3h) using two radiant heat exposure devices (**Figure** **4**a). Notable differences between the garment's initial and activated states under ambient and elevated temperatures were discerned, with noticeable alterations in thickness distinctly represented in Figure 3i,j respectively.

The performance of our novel SRT was evaluated through thermal tests involving radiant heat exposure and contact with hot surfaces. The radiant heat exposure and hot surface contact tests were designed to mimic conditions encountered in firefighting environments. Firefighters are subjected not only to direct firelight radiation but also to the intense heat of air influenced by fire. They may also interact with various objects during their operations. Thus, the hot surface contact test is vital in evaluating the protective potential of the materials. Typically, a fire environment maintains temperatures from 100 to 300 °C and delivers a heat flux approximately in the range of 1–10 kW m^−^^2^.^[^ ^32^ ^]^ Specifically, we set the outer layer temperatures to 117 °C for the radiant heat exposure test and to 120 °C for the hot surface contact test. The experimental setup for the radiant heat exposure is depicted in **Figure** **4**a. Compared to the traditional textile (TT), our SRT maintained a lower temperature within its internal layer throughout the testing process (Figure 4b). Upon exposure to radiant heat, the low boiling point fluid within the SRT absorbed heat, transitioned to vapor, and consequently increased the material's thickness, which enhanced the thermal resistance which initially rose and then stabilized once the phase changed from liquid to vapor was complete. In contrast, the TT displayed a more rapid increase in internal temperature, indicating a delay in the accumulation of heat, indicating a lag in heat prevention. Human skin is known to experience pain at temperatures ≈44 °C, and burns can occur if the temperature on the skin's surface surpasses this threshold. Temperatures reaching ≈56 °C can cause second‐degree burns that might result in permanent damage. Therefore, the “pain alarm time”—the duration from the onset of pain to the infliction of a second‐degree burn—is critical, as it allows individuals sufficient time to evacuate hazardous areas.^[^ ^32^ ^]^ To evaluate safety, we measured the time taken for the inner layer to reach 44 (t_44_) and 56 °C (t_56_) along with the final temperature of the inner layer (*T*_fa_) in Figure 4c. The adoption of SRT significantly prolonged the time of the inner layer to reach both 44 and 56 °C, particularly the latter, which was notably delayed. Moreover, the *T*_fa_ reduced by 22.8%, from 65.9 to 50.9 °C, reinforcing the effectiveness of SRT. In fact, t_56_ was not reached, as indicated by the *T*_fa_ remaining below 56 °C. Additionally, we conducted simulations to analyze the temperature and velocity distribution within the SRT, the results of which are illustrated in Figure 4d. This simulation suggested that incorporating a knitted thermal liner could segment the internal air gap into smaller compartments, thereby diminishing thermal convection. This can be substantiated by the thermal resistance values displayed in Table S1 (Supporting Information). The samples with STAs enveloped in the knitted thermal liner show higher thermal resistance than those with single STAs directly placed on the liner in a fully inflated state. It is attributed to that when STAs are encased within the knitted thermal liner, the connection clap compartmentalizes the air gap into smaller sections when inflated, resulting in the efficient reduction of convective heat transfer.

We also conducted experiments to assess the response of the fabric when exposed to heated surfaces. For these tests, as detailed in reference,^[^ ^33^ ^]^ we placed the fabric on a hotplate preheated to 120 °C, ensuring the outer layer was in direct contact with the heat source. Heat transfer from the hotplate to the fabric occurred sequentially from the outer layer to the inner layer. This process triggered SMAs embedded within the fabric to expand, thereby increasing the gap between the inner layer and the surface of the hotplate. The experimental setup is illustrated in Figure 4e. The temperature‐time profile, depicted in Figure 4f, reveals that the SRT maintained a significantly lower temperature at the inner layer—exceeding a 10 °C difference—compared to the TT. The temporal variation denoted as t_44_ was relatively minor, suggesting that the SMAs were either not yet activated, or the deformation was minimal upon reaching 44 °C. However, the time difference value of t_56_ was remarkable, indicating full activation of the actuators. Additionally, *T*_fa_ dropped by 24.5%, from 66.1 to 49.9 °C. Simulation results for the temperature and airflow velocity distribution across the SRT are presented in Figure 4h, demonstrating the efficacy and contribution of the engineered knitted thermal liner. We further evaluated thermal comfort through tests measuring thermal resistance and moisture permeability to understand the fabric's insulation properties and its ability to facilitate moisture transport from the human body to the surrounding environment. The sweating guarded hotplate used for this evaluation is shown in Figure 4i. The comparison in Figure 4j indicates that, relative to the TT, the SRT displayed reduced thermal resistance when deflated but exhibited significantly increased thermal resistance once inflated. Compared to the fully deflated SRT, thermal resistance rose by 101.13% (from 0.2394 to 0.4815 K m^2^ W^−1^), surpassing recent thermal protective clothing (refer to Table S3, Supporting Information for this comparison). Notably, the difference in moisture resistance between the deflated and inflated states was no more than 20 Pa m^2^ W^−1^, with the maximum value being below 70 Pa m^2^ W^−1^. These findings support the practical application of the knitted thermal liner for wear comfort and robustness. Lastly, Figure 4l visually presents the variation in textile thickness; the SRT possessed a thinner profile when deflated and a larger one when inflated, compared to the TT.

Furthermore, to examine the thermostability of the materials used in the proposed textile, we carried out both a burning test and a thermogravimetry analysis (TG) test. The outer shell is the layer that comes into direct contact with fire and heat. Its function is to resist external fire or heat without damaging the overall protective function of the suit, that is, fire prevention and thermal insulation. The burning test results indicated that the damaged length of the outer shell was not larger than 1 cm and the afterflame time was not longer than 2 s. The TG test results, illustrated in Figure S10 (Supporting Information), reveal that the outer shell's weight begins to decrease at 250 °C. Meanwhile, the weights of the TPU fabric, knitted thermal liner, and moisture barrier begin to reduce at 325, 372, and 335 °C respectively – all above 250 °C. These findings suggest that these materials possess greater thermostability than the outer shell. Therefore, the materials utilized exhibit robust resilience to high fire ground temperatures. Meanwhile, the breaking strength of the TPU fabric and the bonding strength of the sealing area have been gauged, with details available in Supplementary Materials (Section 8: Mechanical Properties of STA). The breaking load was noted to be 385.77N, while the bonding strength of the STA's sealed area is 312N, ≈81% of the TPU fabric's breaking strength, showing minimal strength loss.

## 3 Conclusion

In this paper, we introduce a soft robotic textile designed for superior, adaptive thermal protection. This self‐actuating textile functions effectively with the reversible phase change of a low‐boiling‐point fluid in response to varying temperatures. This process is markedly enhanced by incorporating a knitted fabric as the thermal liner. The liquid‐to‐vapor transformation acts as a thermal regulator, automatically adjusting to changes in the surrounding environment, thereby alleviating the need for the wearer to layer or shed clothing. Nobly, the fabric system is primarily powered by passive heat obtained from the environment, eliminating the requirement for additional energy sources. The materials employed are not only commercially available at a low cost but are also simple to manufacture using a scalable and straightforward process, paving the way for mass production. Our innovative soft robotic textile demonstrates exceptional performance in safeguarding individuals in extreme and dynamic environmental conditions (Figure S12, Supporting Information).

## 4 Experimental Section

### Materials

The low boiling point fluid used in this paper was Novec 7100 fluid from 3 m, which has a boiling point of 61 °C. This fluid exhibits a range of safety and environmental benefits as it is non‐explosive, non‐flammable, non‐conductive, and possesses low toxicity levels. Its environmental credentials were further enhanced by a low Global Warming Potential (GWP) and zero ozone depletion potential, underpinning its impressive profile in terms of environmental, health, and safety considerations. Consequently, Novec 7100 emerges as an appropriate choice for incorporation into the design of garments for human use. The material specifications described in this paper include heat‐sealed fabric made from 100% nylon woven fabric coated with TPU, having a weight of 134 g m^−^^2^ and a thickness of 0.15 mm. The wicking layer consists of woven cotton fabric with a thickness of 0.65 mm. The outer shell, fabricated from 100% Nomex, weighs 327 g m^−^^2^ and has a thickness of 0.68 mm. The conventional moisture barrier employed consists of a composite of 100% meta‐aramid, PTFE film with a mass per unit area of 89 g m^−^^2^ and a thickness of 0.25 mm. The breathable liquid moisture barrier was a polyester fabric weighing 92 g m^−^^2^ with a thickness of 0.62 mm. The thermal liner was constructed from 100% cotton fabric, with a weight of 263 g m^−^^2^ and a thickness of 3.88 mm, whereas the knitted thermal liner features a knitted fabric made solely from cotton at 752.9 g m^−^^2^ and a thickness of 2.24 mm.

### Thermal Protection Testing Procedures

The radiant heat exposure test was performed utilizing an infrared heater to establish a surface temperature of 117 °C on the exterior shell fabric. To monitor temperature fluctuations, sensors were installed on both the outer and inner surfaces. In the hot surface contact test, the material was placed upon a surface heated to 120 °C to observe variations in the temperature of the inner lining. In both experiments, the fabric's outer layer was positioned in proximity to the heat sources. Specifically for the radiant heat exposure assessment, a radiant heat source covered with thermal protective fabric was situated at a distance of 15 cm from the target, supported by a bracket system. Two sensors were affixed—one on the exterior of the outer shell and another on the interior of the thermal liner to ensure precise measurement of temperature differentials. Regarding the hot surface contact evaluation, the thermal protective fabric sample was laid onto a pre‐heated plate set to a prescribed temperature. A single sensor was strategically located on the surface of the inner layer to continuously track temperature shifts.

### Thermal Comfort Evaluation

The thermal comfort of the fabric was assessed using a YG(B) 606G Textile Thermal and Moisture Tester from Wenzhou Darong Textile Instrument Co., Ltd. This evaluation was conducted in accordance with the standard ASTM F1868. For the tests, the hotplate temperature was set to 35 °C. The thermal resistance tests were carried out in a chamber maintained at 20 °C with 65% relative humidity, while the moisture resistance tests were conducted at 35 °C and 40% relative humidity.

### Statistical Analysis

The dimensions of a single STA are detailed in Figure S3 (Supporting Information). The size of the knitted thermal liner was addressed when introducing the liner. All data were expressed as mean ± standard deviation. All data analyses were carried out using MATLAB code (2021b) and simulations were computed by ANSYS‐FLUENT.

## Conflict of Interest

The authors declare no conflict of interest.

## Supporting information

Supporting Information

Supplemental Movie 1

Supplemental Movie 2

Supplemental Movie 3

Supplemental Movie 4

## Data Availability

The data that support the findings of this study are available in the supplementary material of this article.
