# Supplementary material for: Soft Robotic Textiles for Adaptive Personal Thermal Management
Source: Adv Sci (Weinh). 2024 Mar 26;11(21):2309605. doi: 10.1002/advs.202309605 (PMC11151060; doi:10.1002/advs.202309605)
Supplement: Supplementary file 1 — Supporting Information [file ADVS-11-2309605-s001.pdf]

ADVANCED SCIENCE
Open Access

Supporting Information

for *Adv. Sci.*, DOI 10.1002/advs.202309605

Soft Robotic Textiles for Adaptive Personal Thermal Management

*Xiaohui Zhang, Zhaokun Wang, Guanghan Huang, Xujiang Chao, Lin Ye, Jintu Fan and Dahua Shou**

Supplementary Materials for

Soft Robotic Textiles for Adaptive Personal Thermal Management

Xiaohui Zhang[a,b,c], Zhaokun Wang[a], Guanghan Huang[d], Xujiang Chao[a,e], Lin Ye[f], Jintu Fan [a,b], Dahua Shou[a,b,c]*

[a]Future Intelligent Wear Centre, School of Fashion and Textiles, The Hong Kong Polytechnic University, Kowloon, Hong Kong, 999077, China

[b]Research Centre of Textiles for Future Fashion, The Hong Kong Polytechnic University, Hung Hom, Kowloon, Hong Kong, 999077, China

[c]Research Institute for Intelligent Wearable Systems, The Hong Kong Polytechnic University, Hung Hom, Kowloon, Hong Kong, 999077, China

[d]State Key Laboratory of Precision Electronic Manufacturing Technology and Equipment, Guangdong University of Technology, Guangzhou 510006, China

[e]School of Mechanical Engineering, Northwestern Polytechnical University, Xi'an 710072, China

[f]School of System Design and Intelligent Manufacturing (SDIM), Southern University of Science and Technology, Shenzhen 518055, China

*Corresponding author.

E-mail address: dahua.shou@polyu.edu.hk

The PDF file includes:

- Fabrication of STAs
- Theoretical model of thermally-adaptive actuation
- Internal pressure of STAs and their characterization
- Amount of low boiling point fluid
- Wicking fabric property
- Assembly of soft robotic textiles
- Theoretical and experimental analysis of the knitted thermal liner
- Thermostability of the proposed textile
- Mechanical properties of STA
- Durability of STA
- Comparison of thermal properties between present and recent work
- Design diagram of firefighter suit using soft robotic textiles
- Reference

Other Supplementary Material for this manuscript includes the following:

- Movie S1: Deformation procedure of single STA.
- Movie S2: Demonstration of the cross section of single STA during deformation procedure.
- Movie S3: Leakage test.
- Movie S4: Deformation procedure of fabric clothing under hotplate.

1. Fabrication of STAs

The TPU fabric is an environmentally friendly inflatable material that is commonly applied in the production of lightweight inflatable elements. In this study, it is nylon fabric with a TPU coating on one side (Figure S1a). The nylon fabric provides remarkable tear and tensile strength. TPU is a thermoplastic elastomer that is capable of being melted and processed, possessing exceptional tensile strength and durability. The TPU coating provides both water resistance and airtightness, effectively preventing liquid penetration and air transmission. The heat sealer used is shown in Figure S1b.

The fabrication steps of STAs are illustrated in Figure S1c. 1) Two layers of TPU material were layered with wicking fabric sandwiched in between. 2) The integrated fabric system was sealed using thermal bonding technology with a heat sealer to create multiple long strips of bags. The width of the sealed area was determined by the dimensions of the heat sealer's heater. 3) The low boiling point fluid was injected into the non-sealed area, also known as the air chamber, and absorbed by the wicking fabric. 4) The TPU was promptly sealed in a vacuum to prevent liquid loss by the heat sealer. 5) The STAs that were constructed were cut to match the sealed shape. The air chamber is a sealed-off space designed for facilitating the conversion of the low boiling point fluid between liquid and vapor.

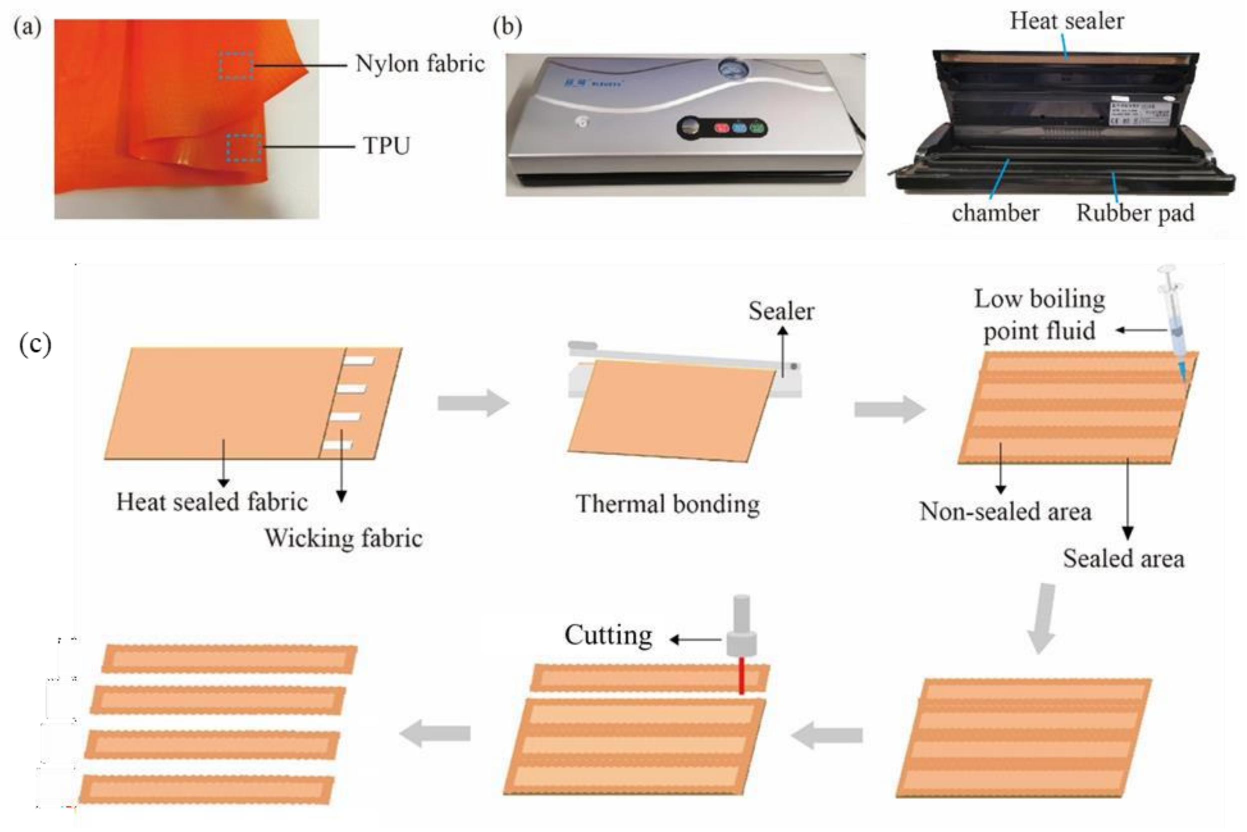

Figure S1. Fabrication of STAs. (a) TPU fabric with one side coated by TPU. (b) shows the heating sealer. The heating sealer has an air chamber to achieve vacuum packing of STA. (c) Fabrication process of STAs. Thermal bonding technology is applied to seal the edges of the heat-sealed fabric, avoiding liquid or vapor leakage. The surface with TPU coating was sealed together by thermal bonding technology using heat sealer, which may allow for larger-scale industrial manufacture.

2. Theoretical model of thermally-adaptive actuation

According to the liner actuation modes proposed by Koya Narumi for Pouch Motors,[1-3]the STA is in a thin sheet shape with no pressure as shown in Figure S2a. In order to facilitate the analysis of STA deformation, the following parameters are all for the air chamber, without considering the sealed edges. When there is a passive pressure *(P)* inside the STA, the geometry is an airfoil shape with cylindrical surfaces on the top and the bottom layer due to the extension of the top and bottom surface, and the contraction of the two edges as well as the rotation of the points, which is shown in Figure S2b. Figure S2c demonstrates the parameters of the activated STA, whose cross-section can be assumed as a ellipse, and the front view of the ellipse is shown in Figure S2d. The minor axis of the ellipse *(H)* can be seemed as the height of the STA.

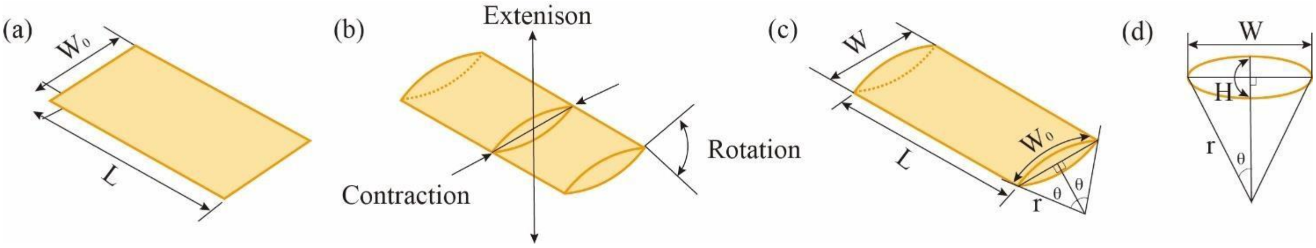

Figure S2. (a) STA in a thin sheet. (b) Dimension change of the STA. (c) Model of the single STA in activated state.(d) Front view of the cross-section of STA.

When the temperature rises, the liquid evaporates, increasing the inner pressure and the volume of STA *(V)*. At the same time, the width *(W)*, the height *(H)* and the radius of the curvature *(r)* of the cylindrical surfaces are also changed. It is assumed that the TPU fabric is inextensible with zero bending stiffness, and the length of STA *(L)* is constant during the deformation process. W_0 is the original width of the STA when it is fully deflated, θ is the central angle of the circular segment, and W is the width of

cross-section of STA.

The width of the STA has already been analyzed theoretically as

$$W(\theta) = W_0 \frac{sin\theta}{\theta} \quad \text{(S-1)}$$

The height of the STA can be derived as

$$H(\theta) = \frac{W_0(1 - cos\theta)}{\theta} \quad \text{(S-2)}$$

The volume of the STA can be derived as

$$V(\theta) = \frac{{W_0}^2 L}{2} \left(\frac{\theta - \cos\theta \sin\theta}{\theta^2}\right) \quad \text{(S-3)}$$

The theoretical minimum contraction ratio of the STA occurs when the STA is thin without liquid evaporation. The height of the STA is equal to the thickness of fabric layers. While the maximum contraction ratio of the STA can be achieved when θ is $\frac{\pi}{2}$. And the cross-section of the STA can be assumed as a circle. Under that condition, the maximum height of single can be derived before fabrication according to the original width *(W_0)* as the circumference is twice the original width.

Based on the analysis above, the air chamber of the STA measured 30mm × 300mm in size and was capable of achieving a maximum height of 19.1mm, demonstrated in Figure S3a. The relationship between the original width in the fully deflated state and the maximum thickness of the STA was also shown in Figure S3c. The width of the wicking fabric used was 28mm and its length was 300mm, as shown in Figure S3b. Overall, the smaller width of the wicking fabric ensured that it could be inserted easily into the air chamber of the STA.

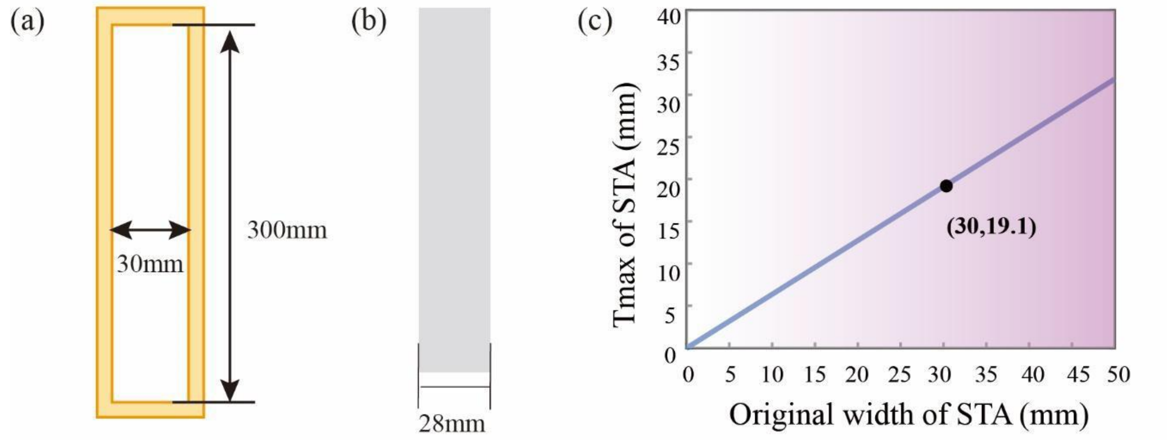

Figure S3. (a) The size of the STA. (b) The size of the wicking fabric. (c) The relationship between the original width in fully deflated state and the maximum height of the STA in fully inflated state.

3. Internal pressure of STAs and their characterization

According to the product information from 3M, the variation of vapor pressure with temperature for 3M™ Novec™ 7100 Engineered Fluid can be calculated using the following formulas:

$$\ln P = 22.415 - 3641.9[1/(t + 273)] \qquad \text{(S-4)}$$

P=Vapor Pressure in Pascals

t = Temperature in °C

The boiling point of the fluid is 61°C, the corresponding pressure is near 10^5 Pa, equal to one atmosphere. Based on the pressure test results, the sealed area can withstand a pressure of 1.96MPa at a corresponding temperature of 185.5℃. This indicates that the maximum operating temperature for the smart thermal actuators is 185.5℃. If the temperature within the actuators exceeds 185.5℃, these devices could potentially explode, leading to the release of vapors. According to the operating temperature, 1mL of low-boiling point of fluid was injected into STA. If the volume is less than 1mL, the STA may not be fully inflated when the temperature approached its boiling point. While if the volume is more than 1mL, the STA may explode under the maximum operating temperature due the excessive internal pressure. The relationship between temperature and the pressure was shown in Figure S4.

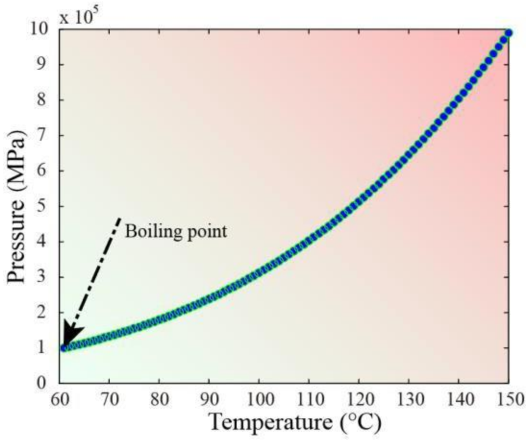

Figure S4. The relationship between temperature and the pressure.

4. Amount of low boiling point fluid

As mentioned above, the STA can be inflated since the large amount of vapor released when the liquid evaporates. Therefore, the volume of the liquid injected into STA is significant. The STA may not fully inflate due to the small amount of liquid. While if the liquid is too much, the weight and the cost of the STA would increase. On the other hand, the large volume of liquid also leads to large amount of vapor, which may bring higher internal pressure, exceeding the maximum pressure of STA. Thus, the minimum volume of the liquid V_l [m^3] required should be calculated.

Assuming that the maximum theoretical volume of the STA filled with vapor is V [m^3]. The maximum amount of vapor in a single STA is n_v [mol]. According to the state equation PV = nRT,

$$n_v = \frac{P}{RT}V \tag{S-5}$$

The mass of the liquid m [kg] can be derived as

$$m = M n_v \times 10^{-3} \tag{S-6}$$

Where M [g/mol] is the molar mass of the liquid. The density of the liquid is ρ [kg/m^3], thus

$$\begin{aligned} V_l &= \frac{m}{\rho} \\ &= \frac{M n_V \times 10^{-3}}{\rho} \\ &= \frac{M}{\rho}\frac{P}{RT}V \times 10^{-3} \end{aligned} \tag{S-7}$$

According to Eq.(S-3), the maximum volume of STA appears when θ is $\frac{\pi}{2}$. Therefore,

$$\begin{aligned} V_l &= \frac{M}{\rho}\frac{P}{RT}V\left(\frac{\pi}{2}\right) \times 10^{-3} \\ &= \frac{M}{\rho}\frac{P}{RT}\frac{{W_0}^2 L}{\pi} \times 10^{-3} \end{aligned} \tag{S-8}$$

According to the product information from 3M, M=250, ρ=1520. For STA, W_0=0.03m, L= 0.3m. The boiling point of the liquid is 61°C. Thus, the temperature should be at least 334K to drive the actuator and the pressure is 99807.64Pa under this temperature. R is 8.31J/(mol・K). Given the aforementioned parameters, it can be inferred that the minimum volume of the liquid required for the proposed STA used is calculated as 0.51mL. In order to guarantee the totally inflation of the STA, the volume of the liquid injected in this paper is 1mL.

5. Wicking fabric property

The wicking fabric applied is shown in Figure S5a, which is made by cotton with woven structure. In order to test the wicking performance of the cotton fabric, a transparent bag with low boiling point fluid (5mL) was prepared firstly, shown in Figure S5b. Figure S5c&d showed that the liquid in the bag was reduced to a small amount after adding the cotton fabric as most of the liquid was absorbed by the wicking fabric.

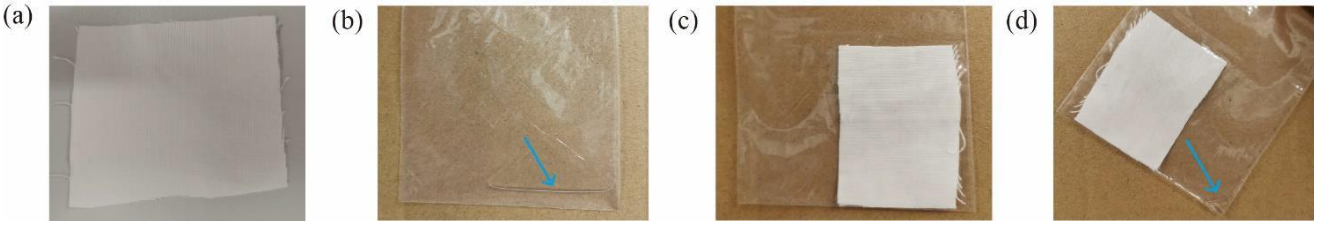

Figure S5. (a) The cotton fabric used as the wicking fabric. (b) The low boiling point in a transparent bag. (c & d) The wicking performance of the wicking fabric. The cotton fabric can absorb the liquid. Upon tilting the bag, the remaining liquid is significantly reduced.

6. Assembly of soft robotic textiles

Under typical circumstances, the STAs can be incorporated equidistantly between the moisture barrier and the thermal liner and affixed to the thermal liner as illustrated in Figure S6a. As the temperature rose, the fluid underwent a significant phase transition from liquid to vapor, resulting in a substantial increase in volume of STA. The inflated STA brought the moisture barrier to move away from the thermal liner. Thus, an air gap was created between them to prevent heat transfer from the exterior. The cross section of the fabric systems could be seen from Figure S6c. However, the limitation of this structure is apparent as the STAs exhibit instability. And the height of the air gap was approximately equal to the height of the STA. According to the previous literature, the thermal insulation can be enhanced when the air gap thickness increases.[4-5] However, the heat convection may occur with the further increase of the air gap, which can weaken the insulating effect of air gap. To address those issues, our solution involves implementing a knitted thermal liner equipped with channels that can be used to mount STAs, as illustrated in Figure S6b. The STAs were inserted into the channels of knitted thermal liner and connected by connection clap. When the temperature is low, the STAs were flat, and all the fabric layers contacted with each other. As the temperature rose, the liquid began to evaporate,

leading to inflation of STA. The channels were isolated, and an air gap was established concurrently. And the cross section of the textiles from this approach was depicted in Figure S6d. As displayed in Figure S6e & f, the ideal unit of the cross section of the textiles from the two approaches were selected. The figures showed that the application of the knitted thermal liner to wrap STAs can divide the air gap into smaller parts, aiming to weaken the thermal convection. The images of the textile were showed in Figure S6g.

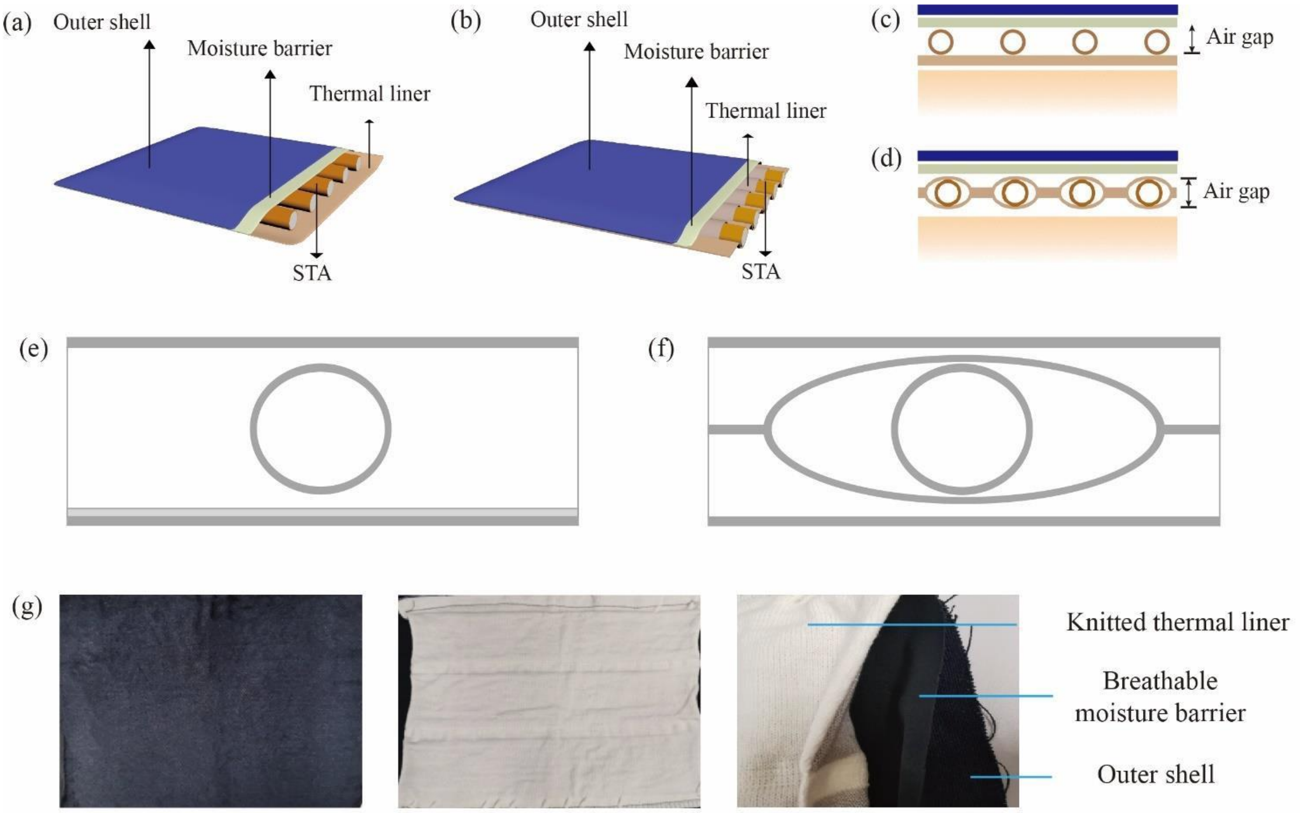

Figure S6. (a) The textile with STA between the moisture barrier and the thermal liner. (b) The proposed SRT with STA inside the channel. (c) The cross section of the textile mentioned in (a). (d) The cross section of the SRT. (e) The unit of the cross section of the textiles with STAs under the thermal liner. (f) The unit of the cross section of the textiles with STAs wrapped by the thermal liner. (g) The image of SRT: the outer shell, the inner layer of the knitted thermal liner and the three layers of the SRT.

7. Theoretical and experimental analysis of the knitted thermal liner

Since air gap inside the clothing plays an important role in heat transfer performance, quantifying the size of air gaps in thermal protection clothing is essential. According to previous research, the air gap

between fabric layers can effectively improve the thermal insulation properties as the lower thermal conductivity of air than conventional fibers applied in thermal protective clothing. However, the insulation effect of the air gap can be weakened due to the natural convection occurring when the size of the air gap is large enough. In order to reduce the influence led by natural convection, a knitted thermal liner with channels was developed to wrap the actuators. As most of the previous literature carried out analysis for the widths of air gap ranging from 6.4mm to 19.1mm, the present study would discuss the clothing with different air gap width of 6.4mm, 12.7mm, 19.1mm and 25.4mm by numerical modeling programmed with ANSYS-FLUENT. The simulation setup was illustrated in Figure S7.

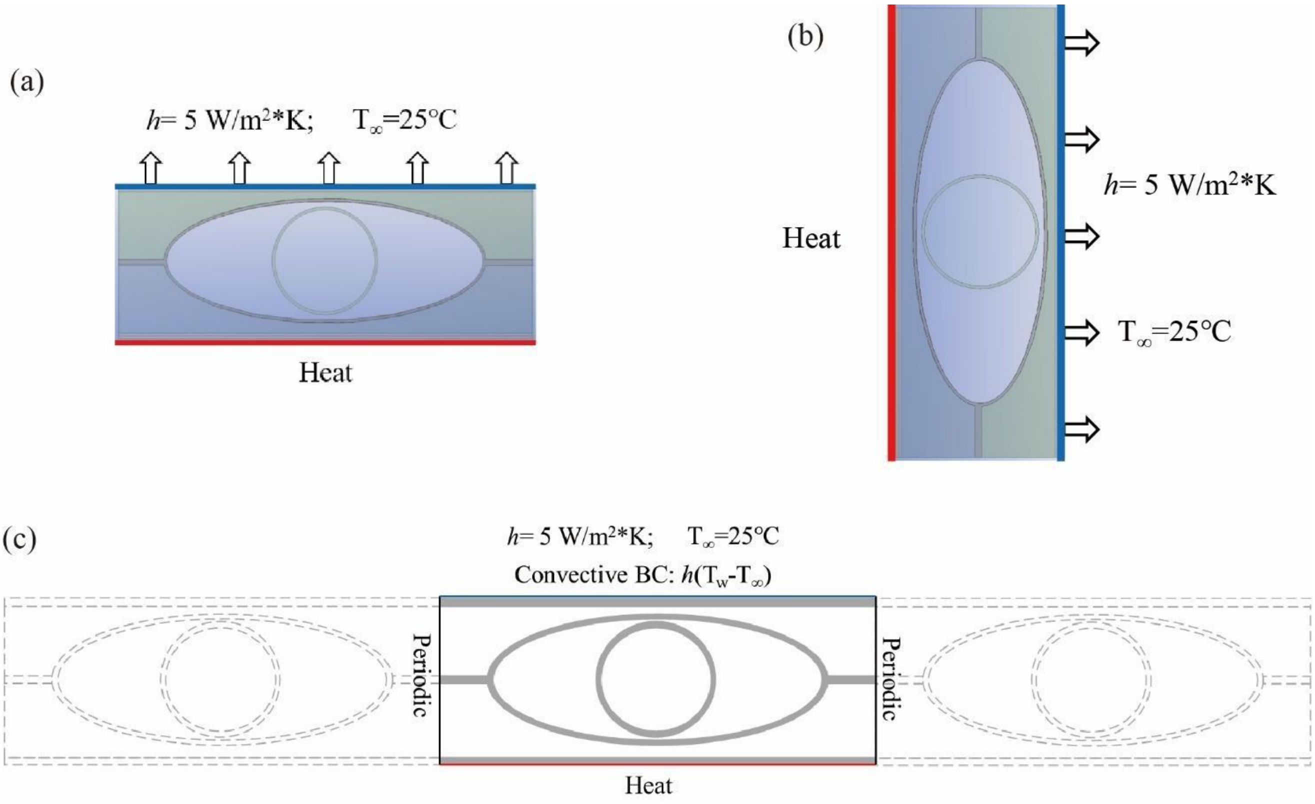

Figure S7. The simulation setup. (a) The setup for horizontal direction. (b) The setup for vertical direction. (c) The Schematic for simulation configuration.

Figure S8a showed the results of the clothing with knitted thermal liner with different air gap under horizontal direction. When the original width of the channel is 60mm, the clothing with 19.1mm air gap demonstrated lower temperature than the 6.4mm and 12.7mm due to the larger air gap. However, when the air gap width is 25.4mm, the temperature was much higher. As mentioned above, the possible

reason is that the larger air gap led to stronger thermal convection, enhancing the heat transfer. Then, in order to discuss the relationship between the channel width of the knitted thermal liner and the heat transfer, another two types of width were selected. When the original width of the channel is 35mm, it can wrap the actuator tightly. The 87mm-original width of the channel, which is the maximum length in the unit, indicates that there is no connection clap between channels. As shown in Figure.S8b, the maximum and the proposed channel length illustrate much lower temperature. This could be explained from the simulated velocity variations between the air gap in Figure S8d-e. When the actuators are wrapped tightly, the air gap of the connection part is larger, resulting in stronger heat convection, shown in Figure 8d. While the maximum and the proposed channel length showed weaker thermal convection, displayed in Figure S8e & f separately. Actually, when the width of the channel is much larger than the actuator, the supporting force provided by the inflated actuator may not be enough to separate the edge of the channel. The ideal structure mentioned in Figure S8e may not exist. Therefore, this was not adopted. Additionally, one case with the actuator set on the knitted thermal liner was also calculated. Figure S8c showed that the actuators wrapped by the knitted thermal liner had lower temperature, indicating the efficiency of the proposed structure. Figure S8g showed the velocity variations when the STAs were put on the thermal liner. Compared with STAs wrapped by the channels, the velocity was much stronger, leading to the fast heat transfer. Based on the simulated results, it can be concluded that the proposed structure of the SRT has higher thermal insulation performance.

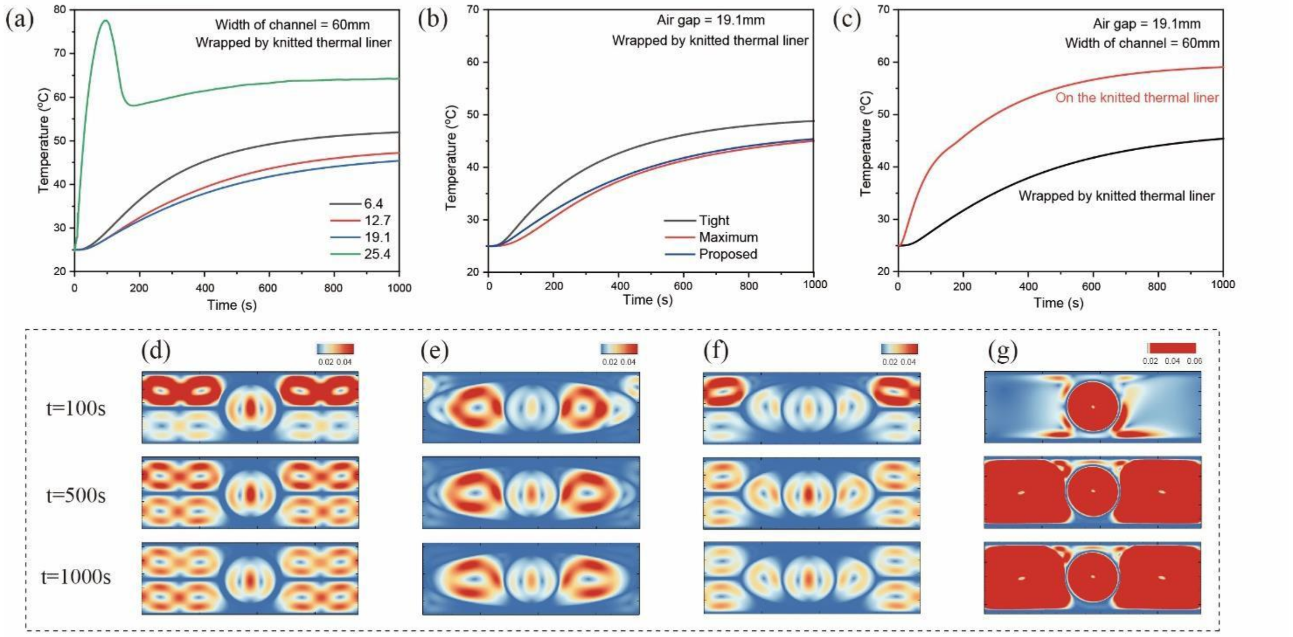

Figure S8. (a) The relationship between exposure time and temperature of the SRT with different air

gap. (b) The relationship between exposure time and temperature of the SRT with width of channels. (c) The relationship between exposure time and temperature of the textiles with different STAs position. (d) The simulated velocity variations at 100s, 500s and 1000s when the channels wrap the actuators tightly. (e) The simulated velocity variations at 100s, 500s and 1000s when the channels reach the maximum length. (f) The simulated velocity variations at 100s, 500s and 1000s under the proposed length. (f) The simulated velocity variations at 100s, 500s and 1000s when the STAs are put on the thermal liner.

In most tests, the air gap of the clothing is horizontal. However, most of the air gap in fabric systems are vertical. Therefore, the model with air gap in vertical orientation was also conducted. The simulated results were shown in Figure S9. The orientation of the air gap in clothing influences the convective heat transfer directly through the air gap due to the buoyancy, which occurs when there is a temperature difference in fluid. Therefore, the horizontal and vertical air gap were both simulated. Figure S9a showed the horizontal air gap, while Figure S9b showed the vertical air gap. As shown in Figure S9c, the fabric with actuators wrapped by knitted thermal liner demonstrated lower temperature. And they had smaller temperature difference even when the orientation of air gap is different.

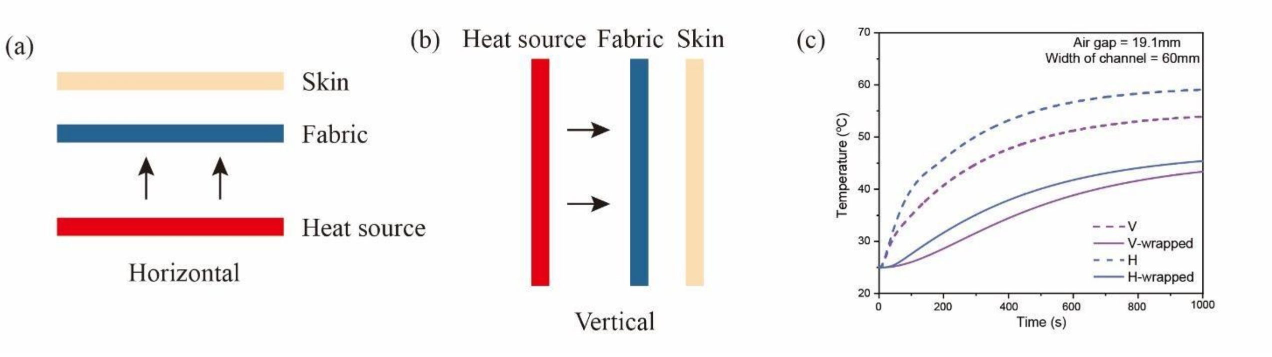

Figure S9. (a) The setup of the experiment in horizontal direction. (b) The setup of the experiment in vertical direction. (c) The comparison of the results when the STAs and the textiles were set in different directions. V and H mean that the STAs were put on the knitted thermal liner and the SRT in vertical and horizontal direction separately. V-wrapped and H-wrapped mean that the STAs were wrapped by the knitted thermal liner and the SRT in vertical and horizontal direction separately.

To validate the hypothesis that wrapping STAs with the thermal liner reduces convective heat transfer among fabrics, we analyzed the thermal resistance of samples with STAs located on the liner, specifically between the liner and the moisture barrier. The experimental findings are detailed in Table S1.

Table S1. Variation of fabric thermal resistance (K·m²/W) across different constructs.

State	Fully deflated	Fully inflated
STA on the thermal liner	0.28	0.378
STA wrapped by the thermal liner	0.2909	0.4507

8. Thermostability of the proposed textile

In order to test the thermostability of the proposed textile, the burning test and TG test were both conducted to study the thermostability of those materials. The TG results for the proposed textile can be seen in Figure S10.

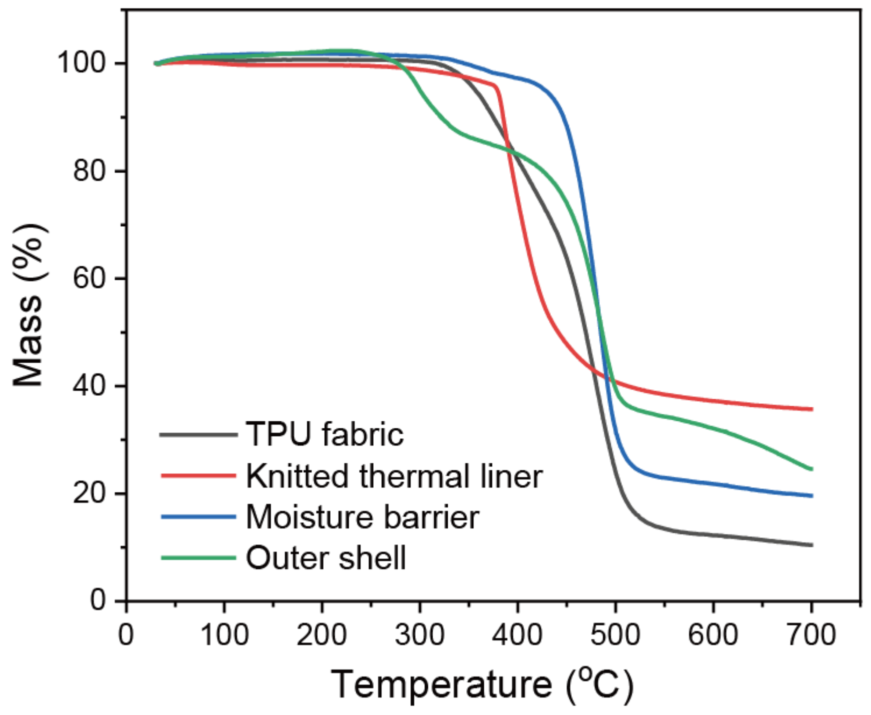

Figure S10. TG results of the fabrics used for the proposed textile.

9. Mechanical properties of STA

In order to test the mechanical properties of STA, Breaking and bonding strength tests were conducted under the *D5035 Textile Breaking Strength/Elongation Strip Method* by Instron 4411. The distance between the clamps is set as 75mm. The breaking extension was 27.16mm and the breaking load was 385.77N. The bonding strength of the sealed area of STA 312N, which is approximately 81% of the breaking strength of TPU fabric. The fabrics before and after tests were illustrate in Figure S10b-c.

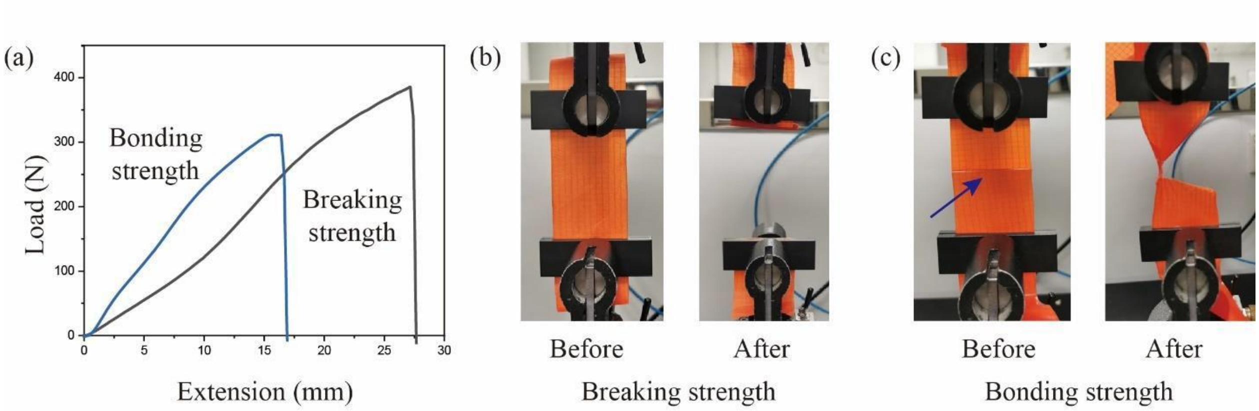

Figure S11. Breaking and bonding strength test. (a) Breaking and bonding strength test results; (b) TPU fabric before and after breaking strength test. (c) Sealed TPU fabric before and after bonding strength test.

10. Durability of STA

To assess the STA's durability, we conducted washing tests under the modified standard: ISO 6330:2021. For the washing trials, we fabricated two small STAs and washed them repeatedly, monitoring the weight fluctuations as a key determinant of durability. As illustrated in the table below, the alterations in the sample weights were minimal, suggesting this change may be negligible.

Table S2. Variation in sample weights throughout the washing tests

Washing time		0	1	2	3	4	5
Weight (g)	1#	1.9769	1.9874	1.9909	1.9914	1.9905	1.9851
	2#	1.8229	1.8316	1.8325	1.8321	1.8302	1.8189

11. Comparison of thermal properties between present and recent work

This research utilized radiant heat exposure tests, hot surface contact tests, and sweating guarded hotplate methods for evaluating thermal protective clothing. However, due to the differing test conditions of radiant heat exposure and hot surface contact tests, comparisons can be unreliable; hence, we selected the sweating guarded hotplate under uniform conditions for comparison.

In previous research [6], a smart fabric system with a shape memory layer was proposed for improved thermal protection. Four samples were designed, one without the shape memory layer (C1) served as a control while others (C2, C3, C4) had different shaped structures. Their thermal resistance is shown in Table S3. Compared to the control, C2, C3, and C4's thermal resistance increased by 90.32%, 91.13%, and 76.61% respectively.

In our study, the SRT in its fully deflated state serves as the control sample. The SRT's thermal resistance when fully inflated rose by 101.13%, superior to the previously mentioned textile, indicating its superior adaptive thermal insulation properties.

Table S3. Comparison of thermal resistance (K·m²/W) between the textiles proposed in our research and those from a recent paper.

Samples	C1	C2	C3	C4	SRT-D	SRT-I
Thermal resistance	0.124	0.236	0.237	0.219	0.2394	0.4815
Increased ratio	0	90.32%	91.13%	76.61%	0	101.13%

12. Design diagram of firefighter suit using soft robotic textiles

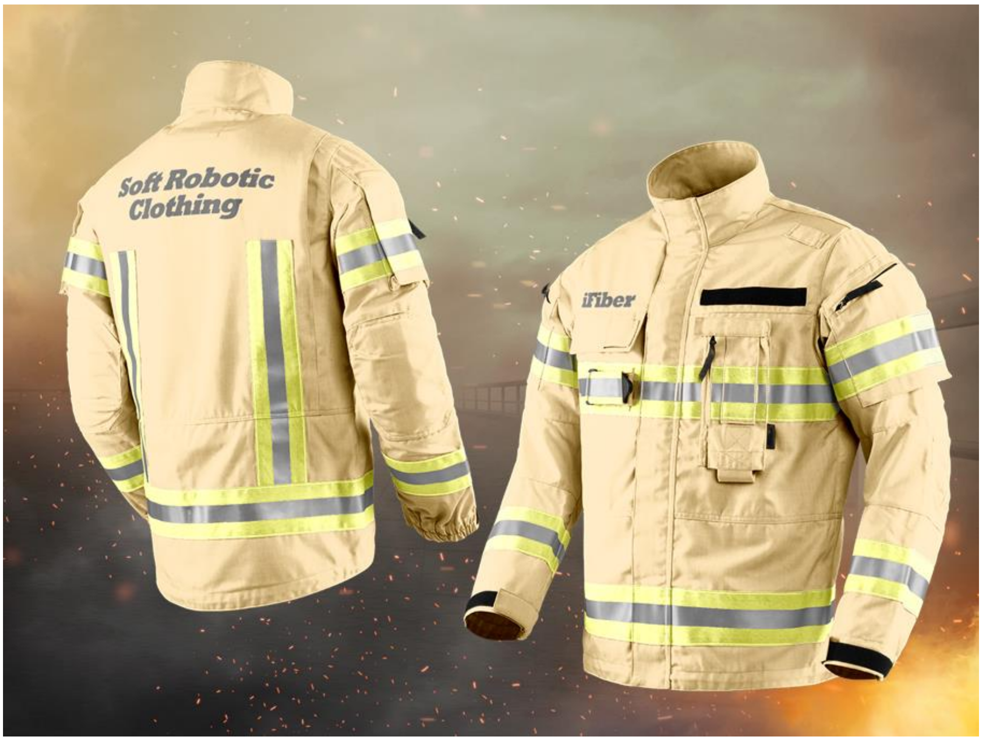

Figure S12. A design diagram of a firefighter suit using soft robotic textiles.

Reference S1

[1] K. Narumi, H. Sato, K. Nakahara, Y. ah Seong, K. Morinaga, Y. Kakehi, R. Niiyama, Y. Kawahara, *IEEE Robotics and Automation Letters* **2020**, 5, 3915.

[2] R. Niiyama, D. Rus, S. Kim, presented at 2014 IEEE International Conference on Robotics and Automation (ICRA) **2014**.

[3] R. Niiyama, X. Sun, C. Sung, B. An, D. Rus, S. Kim, *Soft Robotics* **2015**, 2, 59.

[4] M. Deng, Y. Wang, P. Li, *International Journal of Clothing Science and Technology* **2018**, 30, 246.

[5] C. M. Sawcyn, D. A. Torvi, *Textile Research Journal* **2009**, 79, 632.

[6] L. Wang, M. Pan, Y. Lu, W. Song, S. Liu, J. Lv, *Materials & Design* **2022**, 221: 110922.
